# Endothelial Progenitor Cell Response to Acute Multicomponent Exercise Sessions with Different Durations

**DOI:** 10.3390/biology11040572

**Published:** 2022-04-09

**Authors:** Suiane Cavalcante, Manuel Teixeira, Ana Duarte, Miriam Ferreira, Maria I. Simões, Maria Conceição, Mariana Costa, Ilda P. Ribeiro, Ana Cristina Gonçalves, José Oliveira, Fernando Ribeiro

**Affiliations:** 1 Research Centre in Physical Activity, Health and Leisure, Faculty of Sport, University of Porto, 4099-002 Porto, Portugal; suiane.lima@hotmail.com (S.C.); joliveira@fade.up.pt (J.O.); 2 Institute of Biomedicine—iBiMED, Department of Medical Sciences, University of Aveiro, 3810-193 Aveiro, Portugal; manuelteixeira@ua.pt; 3 Unidade Cuidados na Comunidade Cubo Mágico da Saúde, ACES Baixo Vouga, 3800-120 Aveiro, Portugal; acduarte3@arscentro.min-saude.pt (A.D.); mzferreira@arscentro.min-saude.pt (M.F.); misimoes3@arscentro.min-saude.pt (M.I.S.); mcconceicao@arscentro.min-saude.pt (M.C.); 4 Câmara Municipal de Oliveira do Bairro—Projeto Não Fique Parado, 3800-120 Aveiro, Portugal; oliveira_mariana@msn.com; 5 Cytogenetics and Genomics Laboratory, Institute of Cellular and Molecular Biology, Faculty of Medicine (FMUC), University of Coimbra, 3004-531 Coimbra, Portugal; iribeiro@uc.pt; 6 Institute for Clinical and Biomedical Research (iCBR), Center of Investigation on Environment Genetics and Oncobiology (CIMAGO), Faculty of Medicine (FMUC), University of Coimbra, 3004-531 Coimbra, Portugal; 7 Institute for Clinical and Biomedical Research (iCBR)—Group of Environment, Genetics and Oncobiology (CIMAGO), Faculty of Medicine (FMUC), Center for Innovative Biomedicine and Biotechnology (CIBB), University of Coimbra, 3004-531 Coimbra, Portugal; acgoncalves@fmed.uc.pt; 8 Laboratory of Oncobiology and Hematology, University Clinic of Hematology, Faculty of Medicine (FMUC), University of Coimbra, 3004-531 Coimbra, Portugal; 9 Laboratory for Integrative and Translational Research in Population Health (ITR), University of Porto, 4099-002 Porto, Portugal; 10 Institute of Biomedicine—iBiMED, School of Health Sciences, University of Aveiro, 3810-193 Aveiro, Portugal

**Keywords:** exercise, vascular health, aging, endothelial progenitor cell, flow cytometry

## Abstract

**Simple Summary:**

Reports suggest that an acute bout of exercise promotes the mobilization of endothelial progenitor cells from bone marrow, increasing the circulating levels of endothelial progenitor cells. The impact of the intensity of acute exercise on the circulating levels of endothelial progenitor cells was previously described. Yet, the question of whether the duration of the exercise session impacts the mobilization of endothelial progenitor cells has not been tested among adults with cardiovascular risk factors. In this study, a 30 min session vs. a 45 min session promoted a significant increase in the circulating number of endothelial progenitor cells. The findings show a multicomponent exercise session of only 30 min has the potential to induce beneficial effects for vascular health.

**Abstract:**

It is widely accepted that exercise training has beneficial effects on vascular health. Although a dose-dependent relation has been suggested, little is known about the effects of different exercise durations on endothelial markers. This study aimed to assess the effect of single exercise sessions with different durations in the circulating levels of endothelial progenitor cells (EPCs) and endothelial cells (CECs) among adults with cardiovascular risk factors. Ten participants performed two multicomponent exercise sessions, one week apart, lasting 30 and 45 min (main exercise phase). Before and after each exercise session, blood samples were collected to quantify EPCs and CECs by flow cytometry. The change in EPCs was significantly different between sessions by 3.0% (95% CI: 1.3 to 4.7), being increased by 1.8 ± 1.7% (*p* = 0.009) in the 30 min session vs. −1.2 ± 2.0% (*p* > 0.05) in the 45 min session. No significant change was observed in CECs [−2.0%, 95%CI: (−4.1 to 0.2)] between the sessions. In conclusion, a multicomponent exercise session of 30 min promotes an acute increase in the circulating levels of EPCs without increasing endothelial damage (measured by the levels of CECs) among adults with cardiovascular risk factors.

## 1. Introduction

Aging is associated with vascular endothelial cell senescence and endothelial dysfunction [1], which, in turn, are related to cardiovascular disease development and progression [2,3]. Endothelial cells are considered potential targets for the prevention and treatment of atherosclerotic vascular diseases [4]. Endothelial progenitor cells (EPCs) are bone-marrow-derived cells that play important roles in endothelial cell repair, vascular integrity, and neovascularization [5,6]. EPCs are circulating precursors of endothelial cells that, when attracted to sites of endothelium damage and ischemia tissues [7,8], could promote endothelial repair/regeneration by stimulating the proliferation of mature endothelial cells via a paracrine mechanism, or by incorporating the vessels and differentiating into mature endothelial cells [9,10,11]. Aging [12] and other cardiovascular risk factors [13] are linked to a reduced circulating number and functionality of EPCs; moreover, the circulating number of EPCs seems to predict clinical events in cardiovascular disease [14]. Thus, finding effective strategies of increasing the mobilization of EPC from the bone marrow to the circulation is a promising area of investigation in both primary and secondary prevention of cardiovascular disease. Circulating endothelial cells (CECs) are considered to be an indicator of endothelial injury, because their levels are correlated with endothelial function [15]. The levels of CECs, measured 48 h after acute coronary syndrome, were shown to be an independent predictor of mortality and 1-year major cardiovascular end points at 30 days and 1 year after the acute syndrome [16]. The imbalance between the levels of EPCs and CECs may indicate a lower vascular repair capacity, which makes them potential biomarkers for vascular health [17].

Physical exercise is widely recommended to prevent cardiovascular diseases in adults [18,19] and may counteract the effects of aging on endothelial function [20,21,22]. Exercise seems to maintain endothelial structure/functionality and vascular health, yet the precise mechanisms mediating these effects are yet to be elucidated. Even in patients with heart failure, a disease in which the circulating levels of EPCs are diminished in comparison to an age-matched group [23,24,25], an exercise training intervention lasting from four weeks to six months [26,27,28] is effective in improving EPCs number. Proposed mechanisms include the upregulation of nitric oxide and antioxidant factors, the reduced vascular wall low-grade inflammation, and increased circulating levels of EPCs [8,21]. The long-term beneficial effects of physical exercise occur under cumulative molecular responses to repetitive acute exercise bouts [29]. In this regard, a single exercise session/bout can acutely increase the levels of EPCs [30]; however, few studies have tested whether the mobilization of EPCs from the bone marrow into circulation, and the desquamation of mature endothelial cells, depend on the characteristics (e.g., duration, intensity) of the exercise session [30,31,32,33,34,35]. Among these, only one study tested the acute effects of different durations of exercise in the circulating levels of EPCs among young adults [33].

The duration of the exercise sessions of physical exercise programs among late middle-aged and older adults varies between approximately 9 and 90 min per session [36,37], with 45 min being the most common [36]. There is scarce information on the optimal exercise session duration to optimize the mobilization of EPCs from bone marrow to circulation. Additionally, the number of studies focusing on the response of EPCs and CECs to exercise in late middle-aged and older adults with cardiovascular risk factors is limited. Therefore, this study investigated the effect of single exercise sessions of different durations (30 min vs. 45 min) on the circulating levels of EPCs and CECs among adults with cardiovascular risk factors.

## 2. Materials and Methods

### 2.1. Study Design and Participants

Fifteen physically active adults participating in a primary health care center’s physical activity program were invited to participate in this study. Inclusion criteria: (i) age > 50 years old; (ii) men and women with regular exercise (2 times per week) during the last 6 months; (iii) adults with established cardiovascular disease or at least one cardiovascular risk factor (e.g., diabetes, hypertension, dyslipidemia). Exclusion criteria: any limitation precluding physical efforts, medical history of cancer, or diseases of the hematopoietic system. Five participants did not comply with the eligibility criteria; hence, ten adults participated in this study.

A within-subjects, repeated-measures design was used. All participants completed two multicomponent exercise sessions of different durations (30 or 45 min) on two separate days (one week apart). Both exercise sessions started at the same time of the day (6:00 p.m.). The participants were asked not to engage in any form of exercise on the day of each session. A one-week interval between sessions was used to minimize carry-over effects between exercise sessions. The local ethics committee approved the study (Ref. 174211). All participants provided written informed consent and all procedures were conducted according to the Declaration of Helsinki.

### 2.2. Demographics and Clinical Data

Body weight and height were obtained with a scale and a stationary stadiometer, respectively, and body mass index (kg/m^2^) was calculated. Waist circumference (WC) was measured in the midpoint between the superior iliac crest and the lower margin of the last rib while the participants stood in an erect position with their arms at their sides and their feet close together. Clinical data, medication, and sociodemographic variables (e.g., age, smoking habits) were collected. Resting peripheral blood pressure and heart rate were assessed using an automatic sphygmomanometer (Omron M6, Omron Healthcare, Hoofddorp, The Netherlands) according to the recommendations of the European Society of Hypertension/European Society of Cardiology [38].

### 2.3. EPCs and CECs Quantification by Flow Cytometry

Before and immediately after each exercise session, 3 mL of venous blood was collected into EDTA tubes and treated, according to the manufacturer’s instructions, with TransFix (Cytomark, Caltag Medsystems Ltd., Buckingham, UK) at a 1:5 ratio immediately after collection. Transfix is a cellular antigen stabilization reagent that stabilizes cell populations and permits blood analysis for up to seven days after blood collection [39]. Blood samples were stored in the dark box, at room temperature, until the flow cytometry quantitative assessment of circulating EPCs and CECs (two–three days after blood collection). All the staining and analysis procedures were conducted as previously described [40]. In short, whole blood samples were incubated for 10 min with FcR-blocking reagent to block unwanted binding of antibodies to human-Fc-receptor-expressing cells. All staining procedures were executed at room temperature. Samples were incubated with BV410 CD34 (BD Horizon), PE CD309 (VEGFR-2/KDR; BD Pharmingen), FITC CD144 (BD Pharmingen), BV510 CD45 (BD Horizon), and APC CD133/1 (Miltenyi Biotec), according to the instructions of the manufacturers. After the lysis of the erythrocytes, at least 500.000 CD45^+^ and a minimum of 100 CD34^+^ cells were acquired on a BD FACS Canto II™ system (BD Biosciences, Franklin Lakes, NJ, USA) using BD FACSDiva™ version 6.1.3 software. All samples were analyzed in duplicate. Data were analyzed using Infinicyt^TM^ (Cytognos, Salamanca, Spain). The EPCs were defined as CD45^low^/CD34^+^/CD309^+^/CD133^+^/CD144^−^ cells, and the CECs were defined as CD45^low^/CD34^+^/CD309^+^/CD133^−^/CD144^+^. EPCs and CECs are expressed as a percentage of leukocytes (CD45^+^ cells). The within-day coefficient of variation of EPCs and CECs quantification was <5%.

### 2.4. Exercise Sessions

All participants were familiarized with the type and intensity of exercise. Both exercise sessions took place in the afternoon, between 6:00 and 7:00 pm. Each participant was asked to wear a heart rate monitor (Polar Electro Oy, Kempele, Finland) for monitoring of heart rate throughout the exercise session. The Borg scale was used to assess the rate of perceived exertion (RPE) during the exercise sessions. Each exercise session consisted of three phases: a warm-up phase (5 min of active stretching and mobility exercises of the upper and lower limbs), a main multicomponent exercise phase, and a cool-down phase (composed of 5 min of walking at a light intensity and static stretching). The main multicomponent exercise phase (30 or 45 min) of each session was composed of balance, strength, and aerobic exercise at moderate intensity (12–14 on RPE, 50–60% heart rate reserve). The multicomponent exercise was performed with an interval training design, including periods of walking at light intensity (1 min) with 1 min periods of brisk walking/running intersected with callisthenic exercises (e.g., 1 min light intensity walking–1 min brisk walking/running–1 min light intensity walking–1 min strength exercise–1 min light intensity walking–1 min brisk walking/running, and so on). Each round of the 1 min callisthenic exercises comprised 1 set of 8–12 repetitions of each exercise (i.e., squat, shoulder abduction, front support, and plantar flexion, in sequence). The upper and lower limbs callisthenic exercises were performed with intensity assessed by the participant’s rating of perceived exertion (12–14 on RPE).

### 2.5. Statistical Analysis

All statistical analyses were carried out using the IBM SPSS Statistics 25 software. Normality of the data was tested with the Shapiro–Wilk test. Data are presented as mean ± standard deviation (SD); mean differences are expressed with their two-sided 95% confidence interval (CI). Paired Student *t*-tests were performed for within-session comparisons from baseline to the end of the session. Between-session differences at baseline and in the change from baseline to the end of the session were tested with unpaired Student *t*-tests. The value of significance was set at *p* < 0.05.

## 3. Results

### 3.1. Participant Characteristics

From the 15 participants who were invited to participate in the study, 5 did not comply with the eligibility criteria. Thus, 10 participants agreed to participate and completed both exercise sessions. The participants were mostly women (70%), the mean (SD) age was 67.1 ± 8.6 years, and the most prevalent cardiovascular risk factor was overweight/obesity (60%) followed by dyslipidemia (50%). The heart rate was similar during the exercise sessions (45 min: 114.3 ± 13.4 vs. 30 min: 116.3 ± 18.0 bpm, *p* = 0.64). The characteristics of the participants are summarized in Table 1.

### 3.2. Effects on EPCs and CECs

**Figure 1:**
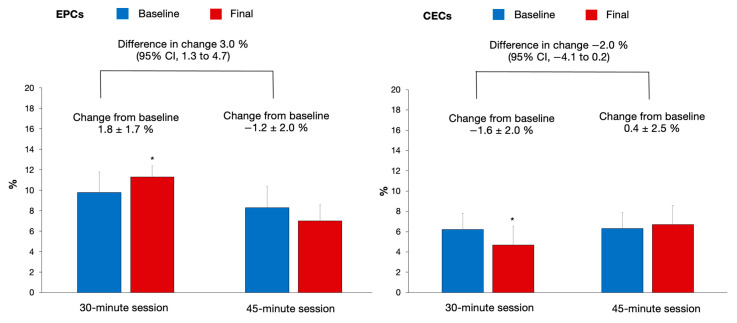
Effects of an acute session of exercise of different durations in the circulating levels of endothelial progenitor cells (EPCs) and endothelial cells (CECs). Data are presented as the number of circulating cells (%) × 10^−3^. * Significant difference from baseline, *p* < 0.05.

The circulating levels of EPCs and CECs were similar before the two exercise sessions (at baseline, *p* > 0.05) (Figure 1). The change in EPCs was significantly different between sessions by 3.0% (*p* = 0.002), being increased by 1.8 ± 1.7% (*p* = 0.009) in the 30 min session vs. −1.2 ± 2.0% (*p* > 0.05) in the 45 min session (Figure 1, Panel EPCs). After the 30 min exercise session, the levels of CECs decreased significantly (*p* = 0.03), while after the 45 min session, the levels of CECs remained unchanged (Figure 1, CECs panel); nevertheless, no significant change was observed between sessions in CECs (Figure 1, CECs panel).

## 4. Discussion

The present study tested the acute effects of multicomponent exercise sessions with different durations (30 and 45 min) on the circulating levels of EPCs and CECs in adults with cardiovascular risk factors. Our results suggest that only the 30 min exercise session significantly enhanced the levels of EPCs and decreased CECs in the peripheral circulation. The mobilization of EPCs from bone marrow was significantly higher in the 30 min session in comparison to the 45 min exercise session.

To the best of our knowledge, the present study is the first to assess the acute effects of two multicomponent exercise sessions with different durations on levels of EPCs and CECs among adults with cardiovascular risk factors. Our findings are partly in agreement with the results of a previous study, assessing the acute effects of two continuous moderate-intensity aerobic exercise sessions with different durations (i.e., 30 and 10 min) on the levels of EPCs among healthy young men [33]. The authors observed that only the 30 min session acutely enhanced the levels of EPCs [33]. Other studies have also showed that 30 min of continuous aerobic exercise in young athletes [32] and resistance exercise in young women [30] increased the circulating levels of EPCs immediately after an exercise session. Regarding the effects of a 45 min exercise session, previous studies showed that a 45 min session of aerobic exercise at 70% VO_2peak_ did not alter the levels of EPCs immediately after exercise in young adults [41] or 60 min after exercise in non-diabetic young men [42]. We anticipated observing an increase in the circulating levels of EPCs after the 45 min exercise session. One potential explanation of our results could be that continuous shear stress for a longer period (45 min vs. 30 min) might increase the activation of endothelial cells to a greater extent, which, in turn, would increase the attraction of EPCs to sites of endothelial activation to stimulate the proliferation of mature endothelial cells or to be incorporated into the vessels, differentiating into mature endothelial cells. This would explain the lack of significant changes in the circulating levels of EPCs immediately after the 45 min session.

Our results showed a decrease in the levels of CECs only after the 30 min session. A previous study [43] found no alterations in the levels of CECs immediately after 30 min of continuous or high-intensity interval exercise in young men. Another study [42] also found no alterations in CECs after 60 min or on the morning after a 45 min session of continuous aerobic exercise. On the other hand, a maximal exercise test increased the levels of CECs in cardiac patients [44,45] but decreased CECs levels at 30 min [45] and 4 h [44] after the exercise test. Taken together, these results suggest that a single 30 or 45 min exercise session does not promote substantial endothelial damage expressed by the CECs count. The possibility of improving vascular repair without increasing the levels of CECs in response to exercise is promising in the context of the prevention of cardiovascular disease.

The exercise characteristics (i.e., mode, intensity, and duration) may have an important impact on vascular response [46]. Indeed, previous studies showed that aerobic exercise protocols (~30 min exercise sessions) at moderate and vigorous intensities increased the number of EPCs [32,33]. Nonetheless, a 10 min session of aerobic exercise at moderate intensity did not change the levels of EPCs in young men [33]; additionally, no alterations were found after 30 min of moderate-intensity continuous aerobic exercise, moderate-intensity interval exercise, or heavy-intensity interval exercise in postmenopausal women [31]. In addition to the characteristics of the exercise session, the level of participants’ physical activity/exercise capacity could explain the difference between our results and the results of previous studies [47]. Nontrained adults submitted to an unhabitual stimuli (i.e., exercise) may present different EPCs and CECs mobilization in comparison with trained subjects. One of the mechanisms that is related to the mobilization of EPCs is exercise-induced shear stress, which leads to the upregulation of nitric oxide [48]. Different modes of exercise can induce different patterns of shear stress [49], which can result in specific responses in vascular function [46,50]. Thus, the quest for the optimal set of exercise session characteristics (i.e., intensity, mode, and duration) to mobilize EPCs from bone marrow to circulation seems a relevant research topic. More studies are needed comparing similar durations of exercise sessions at different intensities and modes in the levels of EPCs and CECs. A strong point of our study is the study of a multicomponent exercise session, since multicomponent exercise is particularly important for late middle-age and elderly adults.

Some limitations should be acknowledged. First, the present study collected the blood samples at a single timepoint after each exercise session, which limited the assessment of the transitory effect over time after both exercise sessions. Second, despite being similar to previous studies in this field [31,41,43], the sample size is also a limitation. We recruited a small sample without performing a previous sample size calculation, which limits the generalizability of our results. This initial study generates data that can be used to determine the sample size in future studies aiming to confirm our findings. Third, the participants did not present a common risk factor, i.e., they presented different cardiovascular risk factors, which could affect the mobilization of EPCs in different degrees. Fourth, further analysis of endothelial damage markers (e.g., assessment of endothelial-derived microparticles [51]), and inflammatory cytokines, such as tumor necrosis factor-alpha [42], could have provided additional information on the effect of exercise on endothelial damage. Finally, since all participants included in this study were trained subjects, it is not clear if the same (or even a more pronounced) effect would be observed in nontrained adults.

## 5. Conclusions

In conclusion, the main results of this study suggest that a shorter bout of multicomponent exercise (30 min) could promote an acute increase in the circulating levels of EPCs without increasing endothelial damage (measured by the levels of CECs) among adults with cardiovascular risk factors.

## Figures and Tables

**Table 1:** Characteristics of the Participants

Age (years)	67.1 ± 8.6
Sex (Male/Female)	3/7
Weight (kg)	65.9 ± 12.4
Height (cm)	158.8 ± 8.8
Systolic blood pressure (mmHg)	129.6 ± 19.4
Diastolic blood pressure (mmHg)	77.7 ± 11.4
Heart rate (bpm)	69.4 ± 7.6
Waist circumference (cm)	93.0 ± 13.2
Body mass index (kg/m^2^)	26.0 ± 3.3
Cardiovascular risk factors (*n*, %)	
Obesity class I	2 (20)
Overweight	4 (40)
Hypertension	3 (30)
Type 2 Diabetes mellitus	2 (20)
Dyslipidemia	5 (50)
Current smoker	1 (10)
Medication (*n*, %)	
Statins	3 (30)
Diuretics	1 (10)
Angiotensin II receptor blockers	1 (10)
Anticoagulants	1 (10)
Calcium channel blockers	1 (10)

Data are presented as means ± SD or number (%).

## Data Availability

The data presented in this study and additional information are available on request to the corresponding author.

## References

[1] Jia G., Aroor A.R., Jia C., Sowers J.R. (2019). Endothelial cell senescence in aging-related vascular dysfunction. Biochim. Biophys. Acta Mol. Basis Dis..

[2] Sun H.J., Wu Z.Y., Nie X.W., Bian J.S. (2019). Role of Endothelial Dysfunction in Cardiovascular Diseases: The Link Between Inflammation and Hydrogen Sulfide. Front. Pharmacol..

[3] Park K.-H., Park W.J. (2015). Endothelial Dysfunction: Clinical Implications in Cardiovascular Disease and Therapeutic Approaches. J. Korean Med. Sci..

[4] Giannitsi S., Bougiakli M., Bechlioulis A., Naka K. (2019). Endothelial dysfunction and heart failure: A review of the existing bibliography with emphasis on flow mediated dilation. JRSM Cardiovasc. Dis..

[5] Asahara T., Murohara T., Sullivan A., Silver M., van der Zee R., Li T., Witzenbichler B., Schatteman G., Isner J.M. (1997). Isolation of putative progenitor endothelial cells for angiogenesis. Science.

[6] Yoder M.C. (2012). Human endothelial progenitor cells. Cold Spring Harb. Perspect. Med..

[7] Wang J.S., Lee M.Y., Lien H.Y., Weng T.P. (2014). Hypoxic exercise training improves cardiac/muscular hemodynamics and is associated with modulated circulating progenitor cells in sedentary men. Int. J. Cardiol..

[8] Guo Y., Ledesma R.A., Peng R., Liu Q., Xu D. (2017). The Beneficial Effects of Cardiac Rehabilitation on the Function and Levels of Endothelial Progenitor Cells. Heart Lung Circ..

[9] Rosenzweig A. (2003). Endothelial progenitor cells. N. Engl. J. Med..

[10] Koutroumpi M., Dimopoulos S., Psarra K., Kyprianou T., Nanas S. (2012). Circulating endothelial and progenitor cells: Evidence from acute and long-term exercise effects. World J. Cardiol..

[11] Zhang M., Malik A.B., Rehman J. (2014). Endothelial progenitor cells and vascular repair. Curr. Opin. Hematol..

[12] Ross M.D. (2018). Endothelial Regenerative Capacity and Aging: Influence of Diet, Exercise and Obesity. Curr. Cardiol. Rev..

[13] Mudyanadzo T.A. (2018). Endothelial Progenitor Cells and Cardiovascular Correlates. Cureus.

[14] Aragona C.O., Imbalzano E., Mamone F., Cairo V., Lo Gullo A., D’Ascola A., Sardo M.A., Scuruchi M., Basile G., Saitta A. (2016). Endothelial Progenitor Cells for Diagnosis and Prognosis in Cardiovascular Disease. Stem Cells Int..

[15] Chong A.Y., Blann A.D., Patel J., Freestone B., Hughes E., Lip G.Y. (2004). Endothelial dysfunction and damage in congestive heart failure: Relation of flow-mediated dilation to circulating endothelial cells, plasma indexes of endothelial damage, and brain natriuretic peptide. Circulation.

[16] Lee K.W., Lip G.Y., Tayebjee M., Foster W., Blann A.D. (2005). Circulating endothelial cells, von Willebrand factor, interleukin-6, and prognosis in patients with acute coronary syndromes. Blood.

[17] Farinacci M., Krahn T., Dinh W., Volk H.-D., Düngen H.-D., Wagner J., Konen T., von Ahsen O. (2018). Circulating endothelial cells as biomarker for cardiovascular diseases. Res. Pract. Thromb. Haemost..

[18] Pelliccia A., Sharma S., Gati S., Bäck M., Börjesson M., Caselli S., Collet J.P., Corrado D., Drezner J.A., Halle M. (2021). 2020 ESC Guidelines on sports cardiology and exercise in patients with cardiovascular disease. Eur. Heart J..

[19] World Health Organization (2010). Global Recommendations on Physical Activity for Health.

[20] Pedralli M.L., Marschner R.A., Kollet D.P., Neto S.G., Eibel B., Tanaka H., Lehnen A.M. (2020). Different exercise training modalities produce similar endothelial function improvements in individuals with prehypertension or hypertension: A randomized clinical trial. Sci. Rep..

[21] Santos-Parker J.R., LaRocca T.J., Seals D.R. (2014). Aerobic exercise and other healthy lifestyle factors that influence vascular aging. Adv. Physiol. Educ..

[22] Bull F.C., Al-Ansari S.S., Biddle S., Borodulin K., Buman M.P., Cardon G., Carty C., Chaput J.P., Chastin S., Chou R. (2020). World Health Organization 2020 guidelines on physical activity and sedentary behaviour. Br. J. Sports Med..

[23] Lopes J., Teixeira M., Cavalcante S., Gouveia M., Duarte A., Ferreira M., Simões M.I., Conceição M., Ribeiro I.P., Gonçalves A.C. (2022). Reduced Levels of Circulating Endothelial Cells and Endothelial Progenitor Cells in Patients with Heart Failure with Reduced Ejection Fraction. Arch. Med. Res..

[24] Samman Tahhan A., Hammadah M., Sandesara P.B., Hayek S.S., Kalogeropoulos A.P., Alkhoder A., Mohamed Kelli H., Topel M., Ghasemzadeh N., Chivukula K. (2017). Progenitor Cells and Clinical Outcomes in Patients With Heart Failure. Circ. Heart Fail..

[25] Chiang C.H., Huang P.H., Leu H.B., Hsu C.Y., Wang K.F., Chen J.W., Lin S.J. (2013). Decreased circulating endothelial progenitor cell levels in patients with heart failure with preserved ejection fraction. Cardiology.

[26] Sandri M., Viehmann M., Adams V., Rabald K., Mangner N., Höllriegel R., Lurz P., Erbs S., Linke A., Kirsch K. (2016). Chronic heart failure and aging—Effects of exercise training on endothelial function and mechanisms of endothelial regeneration: Results from the Leipzig Exercise Intervention in Chronic heart failure and Aging (LEICA) study. Eur. J. Prev. Cardiol..

[27] Van Craenenbroeck E.M., Hoymans V.Y., Beckers P.J., Possemiers N.M., Wuyts K., Paelinck B.P., Vrints C.J., Conraads V.M. (2010). Exercise training improves function of circulating angiogenic cells in patients with chronic heart failure. Basic Res. Cardiol..

[28] Kourek C., Alshamari M., Mitsiou G., Psarra K., Delis D., Linardatou V., Pittaras T., Ntalianis A., Papadopoulos C., Panagopoulou N. (2021). The acute and long-term effects of a cardiac rehabilitation program on endothelial progenitor cells in chronic heart failure patients: Comparing two different exercise training protocols. Int. J. Cardiol. Heart Vasc..

[29] Whyte J.J., Laughlin M.H. (2010). The effects of acute and chronic exercise on the vasculature. Acta Physiol..

[30] Ribeiro F., Ribeiro I.P., Gonçalves A.C., Alves A.J., Melo E., Fernandes R., Costa R., Sarmento-Ribeiro A.B., Duarte J.A., Carreira I.M. (2017). Effects of resistance exercise on endothelial progenitor cell mobilization in women. Sci. Rep..

[31] Harris E., Rakobowchuk M., Birch K.M. (2017). Interval exercise increases angiogenic cell function in postmenopausal women. BMJ Open Sport Exerc. Med..

[32] Krüger K., Alack K., Ringseis R., Mink L., Pfeifer E., Schinle M., Gindler K., Kimmelmann L., Walscheid R., Muders K. (2016). Apoptosis of T-Cell Subsets after Acute High-Intensity Interval Exercise. Med. Sci. Sports Exerc..

[33] Laufs U., Urhausen A., Werner N., Scharhag J., Heitz A., Kissner G., Böhm M., Kindermann W., Nickenig G. (2005). Running exercise of different duration and intensity: Effect on endothelial progenitor cells in healthy subjects. Eur. J. Cardiovasc. Prev. Rehabil..

[34] Schaan B.D., Waclawovsky G., Umpierre D., Figueira F.R., de Lima E.S., Alegretti A.P., Schneider L., Matte U.S., Rodrigues T.C. (2015). A single session of aerobic or resistance exercise modifies the endothelial progenitor cell levels in healthy subjects, but not in individuals with type 1 diabetes. Diabetol. Metab. Syndr..

[35] Krüger K., Pilat C., Schild M., Lindner N., Frech T., Muders K., Mooren F.C. (2015). Progenitor cell mobilization after exercise is related to systemic levels of G-CSF and muscle damage. Scand. J. Med. Sci. Sports.

[36] Zhang Y., Zhang Y., Du S., Wang Q., Xia H., Sun R. (2020). Exercise interventions for improving physical function, daily living activities and quality of life in community-dwelling frail older adults: A systematic review and meta-analysis of randomized controlled trials. Geriatr. Nurs..

[37] García-Hermoso A., Ramirez-Vélez R., Sáez de Asteasu M.L., Martínez-Velilla N., Zambom-Ferraresi F., Valenzuela P.L., Lucia A., Izquierdo M. (2020). Safety and Effectiveness of Long-Term Exercise Interventions in Older Adults: A Systematic Review and Meta-analysis of Randomized Controlled Trials. Sports Med..

[38] Williams B., Mancia G., Spiering W., Agabiti Rosei E., Azizi M., Burnier M., Clement D.L., Coca A., de Simone G., Dominiczak A. (2018). 2018 ESC/ESH Guidelines for the management of arterial hypertension. Eur. Heart J..

[39] Hoymans V.Y., Van Craenenbroeck A.H., Bruyndonckx L., van Ierssel S.H., Vrints C.J., Conraads V.M., Van Craenenbroeck E.M. (2012). TransFix^®^ for delayed flow cytometry of endothelial progenitor cells and angiogenic T cells. Microvasc. Res..

[40] Ahmed F.W., Rider R., Glanville M., Narayanan K., Razvi S., Weaver J.U. (2016). Metformin improves circulating endothelial cells and endothelial progenitor cells in type 1 diabetes: MERIT study. Cardiovasc. Diabetol..

[41] O′Carroll L., Wardrop B., Murphy R.P., Ross M.D., Harrison M. (2019). Circulating angiogenic cell response to sprint interval and continuous exercise. Eur. J. Appl. Physiol..

[42] West D.J., Campbell M.D., Gonzalez J.T., Walker M., Stevenson E.J., Ahmed F.W., Wijaya S., Shaw J.A., Weaver J.U. (2015). The inflammation, vascular repair and injury responses to exercise in fit males with and without Type 1 diabetes: An observational study. Cardiovasc. Diabetol..

[43] Sapp R.M., Evans W.S., Eagan L.E., Chesney C.A., Zietowski E.M., Prior S.J., Ranadive S.M., Hagberg J.M. (2019). The effects of moderate and high-intensity exercise on circulating markers of endothelial integrity and activation in young, healthy men. J. Appl. Physiol..

[44] Mutin M., Canavy I., Blann A., Bory M., Sampol J., Dignat-George F. (1999). Direct evidence of endothelial injury in acute myocardial infarction and unstable angina by demonstration of circulating endothelial cells. Blood.

[45] Boos C.J., Balakrishnan B., Lip G.Y. (2008). The effects of exercise stress testing on soluble E-selectin, von Willebrand factor, and circulating endothelial cells as indices of endothelial damage/dysfunction. Ann. Med..

[46] Dawson E.A., Green D.J., Cable N.T., Thijssen D.H. (2013). Effects of acute exercise on flow-mediated dilatation in healthy humans. J. Appl. Physiol..

[47] Krüger K., Klocke R., Kloster J., Nikol S., Waltenberger J., Mooren F.C. (2014). Activity of daily living is associated with circulating CD34+/KDR+ cells and granulocyte colony-stimulating factor levels in patients after myocardial infarction. J. Appl. Physiol..

[48] Volaklis K.A., Tokmakidis S.P., Halle M. (2013). Acute and chronic effects of exercise on circulating endothelial progenitor cells in healthy and diseased patients. Clin. Res. Cardiol..

[49] Thijssen D.H., Dawson E.A., Black M.A., Hopman M.T., Cable N.T., Green D.J. (2009). Brachial artery blood flow responses to different modalities of lower limb exercise. Med. Sci. Sports Exerc..

[50] Thijssen D.H., Dawson E.A., Tinken T.M., Cable N.T., Green D.J. (2009). Retrograde flow and shear rate acutely impair endothelial function in humans. Hypertension.

[51] Sabatier F., Camoin-Jau L., Anfosso F., Sampol J., Dignat-George F. (2009). Circulating endothelial cells, microparticles and progenitors: Key players towards the definition of vascular competence. J. Cell. Mol. Med..

